# On the Reporting of Odds Ratios and Risk Ratios

**DOI:** 10.3390/nu10101512

**Published:** 2018-10-16

**Authors:** Nelson D. Pace, Jasjit K. Multani

**Affiliations:** 1 Center for Health Sciences, Exponent, Inc., Oakland, CA 94612, USA; 2 Center for Chemical Regulation and Food Safety, Exponent, Inc., Washington, DC 20036, USA; jmultani@exponent.com

**Keywords:** odds ratio, risk ratio, relative risk, epidemiology, logistic regression, statistics

Dear Editor,

It is with great interest that we read the article by Ricci et al. entitled “Maternal and Paternal Caffeine Intake and ART Outcomes in Couples Referring to an Italian Fertility Clinic: A Prospective Cohort” [1]. We congratulate the authors on their research and publication. That said, we have the following two concerns: (1) The misuse of logistic regression given that risk ratios, rate ratios, or hazard ratios could have been calculated from their prospective cohort study design, and (2) the misinterpretation of the resultant odds ratios. 

Ricci et al. conducted logistic regression using data from a prospective cohort study of subfertile couples presenting for assisted reproduction techniques (ART) at a fertility clinic. A result of the logistic regression procedure is the calculation of relative risk estimates known as odds ratios, that is, the odds of exposure (here, parental caffeine intake) among those with the outcome of interest (“ART failure”) divided by the odds of exposure among those without the outcome of interest (“ART success”). This is the same as the ratio of the odds of the outcome of interest among the exposed to the odds of the outcome of interest among the unexposed. Drawing from Table 2 for the context of the article, we find that the odds of a live birth for mothers in the second tertile of caffeine intake is 1.09 (95% confidence interval (CI): 0.79–1.50) times the odds of a live birth for mothers in the first tertile (lowest) of caffeine intake. 

Odds ratios are inherently more difficult to interpret than risk ratios, which can just as easily be calculated in the context of a cohort study such as this by using different statistical procedures, that is, log–binomial regression or Poisson regression with a robust error variance [2,3]. Alternatively, using the available person–time data, the authors could have used Poisson regression or Cox proportional hazards regression to calculate rate ratios or hazard ratios, respectively [4]. Odds ratios approximate risk ratios when the event of interest is “rare” [5,6] (typically, occurring in less than 10% of the study population) [6,7]. However, odds ratios should not be interpreted as risk ratios when the event of interest is common. Furthermore, as the event is increasingly more common, the odds ratio becomes an increasingly poorer approximation of the risk ratio [4,8]. In addition, the farther from the null (i.e., the value 1.0) the odds ratio is, the poorer its approximation of the risk ratio [8].

In Ricci et al., none of the three events of interest was rare: Implantation was unsuccessful in 13.6% of study participants, clinical pregnancy was not reached in 67.6%, and live birth did not occur in 75.8%. Thus, odds ratios are especially inappropriate estimates of risk ratios in this context.

Even if the odds ratio were a good approximation of the risk ratio, the reported results from Ricci et al. would still be in error because the authors label these estimates as *rate* ratios rather than odds ratios or risk ratios. From the article, it is unclear why this was done. Rate ratios tend to be calculated in cohort studies that capture person–time at risk (as opposed to individual persons at risk) or case-control studies with risk-set sampling methods where the calculated odds ratio is a direct approximation of the rate ratio (see Vandenbroucke et al. [9]. for more information on this study design). Ricci et al. could have calculated rate ratios in this study if they had calculated person–time at risk for each subject, but they did not do this. 

In summary, odds ratios are readily calculated from logistic regression models, but such measures of association are ill-suited to cohort studies such as that of Ricci et al., where more readily interpretable risk ratios or possibly rate ratios can be calculated just as easily. Even though the majority of the results of this study indicated no association, this error needs to be prevented in future publications. Should the results have indicated associations of substantial magnitude, the findings might have been erroneously inflated only because odds ratios were reported rather than risk ratios [8].

## References

[1] Ricci E., Noli S., Cipriani S., La Vecchia I., Chiaffarino F., Ferrari S., Mauri P.A., Reschini M., Fedele L., Parazzini F. (2018). Maternal and Paternal Caffeine Intake and ART Outcomes in Couples Referring to an Italian Fertility Clinic: A Prospective Cohort. Nutrients.

[2] Spiegelman D., Hertzmark E. (2005). Easy SAS calculations for risk or prevalence ratios and differences. Am. J. Epidemiol..

[3] Zou G. (2004). A modified poisson regression approach to prospective studies with binary data. Am. J. Epidemiol..

[4] Green M.S., Symons M.J. (1983). A comparison of the logistic risk function and the proportional hazards model in prospective epidemiologic studies. J. Chronic. Dis..

[5] Cornfield J. (1951). A method of estimating comparative rates from clinical data; applications to cancer of the lung, breast, and cervix. J. Natl. Cancer Inst..

[6] Greenland S., Thomas D.C. (1982). On the need for the rare disease assumption in case-control studies. Am. J. Epidemiol..

[7] Knol M.J., Vandenbroucke J.P., Scott P., Egger M. (2008). What do case-control studies estimate? Survey of methods and assumptions in published case-control research. Am. J. Epidemiol..

[8] Davies H.T., Crombie I.K., Tavakoli M. (1998). When can odds ratios mislead?. BMJ.

[9] Vandenbroucke J.P., Pearce N. (2012). Case-control studies: Basic concepts. Int. J. Epidemiol..

